# A Combination of Diffusion and Active Translocation Localizes Myosin 10 to the Filopodial Tip*[Fn FN2]

**DOI:** 10.1074/jbc.M116.730689

**Published:** 2016-08-26

**Authors:** Thomas G. Baboolal, Gregory I. Mashanov, Tatiana A. Nenasheva, Michelle Peckham, Justin E. Molloy

**Affiliations:** From the ‡ Astbury Centre for Structural Molecular Biology and Institute of Molecular and Cellular Biology, University of Leeds, Leeds LS2 9JT and; § The Francis Crick Institute, Mill Hill Laboratory, London NW7 1AA, United Kingdom

**Keywords:** actin, molecular imaging, myosin, single particle analysis, single-molecule biophysics, TIRF

## Abstract

Myosin 10 is an actin-based molecular motor that localizes to the tips of filopodia in mammalian cells. To understand how it is targeted to this distinct region of the cell, we have used total internal reflection fluorescence microscopy to study the movement of individual full-length and truncated GFP-tagged molecules. Truncation mutants lacking the motor region failed to localize to filopodial tips but still bound transiently at the plasma membrane. Deletion of the single α-helical and anti-parallel coiled-coil forming regions, which lie between the motor and pleckstrin homology domains, reduced the instantaneous velocity of intrafilopodial movement but did not affect the number of substrate adherent filopodia. Deletion of the anti-parallel coiled-coil forming region, but not the EKR-rich region of the single α-helical domain, restored intrafilopodial trafficking, suggesting this region is important in determining myosin 10 motility. We propose a model by which myosin 10 rapidly targets to the filopodial tip via a sequential reduction in dimensionality. Molecules first undergo rapid diffusion within the three-dimensional volume of the cell body. They then exhibit periods of slower two-dimensional diffusion in the plane of the plasma membrane. Finally, they move in a unidimensional, highly directed manner along the polarized actin filament bundle within the filopodium becoming confined to a single point at the tip. Here we have observed directly each phase of the trafficking process using single molecule fluorescence imaging of live cells and have quantified our observations using single particle tracking, autocorrelation analysis, and kymographs.

## Introduction

The myosin family of molecular motors is found throughout the eukaryotic kingdom and consists of at least 35 distinct classes (1). The best characterized, class 2, myosins are found in skeletal, cardiac, and smooth muscle cells, where they form filamentous arrays that enable many millions of molecules to work together and generate the large external forces required for muscle contraction. However, of the 39 human myosin genes, only 10 encode muscle myosins; the remaining genes encode three, non-muscle, class 2 isoforms (A, B, and C) and a variety of other myosin types, separated into an additional 11 classes. These myosins are required for a wide range of cellular motilities, including transport of mRNA, proteins, and subcellular organelles; organizing the actin cytoskeleton; cell locomotion; and cytokinesis (2, 3). Here, we have studied the myosin class 10 (M10), which is known to function at the cell periphery and shows distinct localization to the tips of filopodia in mammalian cells (4, 5). It is implicated in the transport of neogenin and the netrin receptor (Deleted in Colorectal Cancer (DCC) (6)), Mena-VASP (7), β-integrins (8), and possibly Sonic Hedgehog (9). Overexpression of M10 in cell lines, such as HeLa and COS-7, induces the formation of numerous and unusually long filopodia (4). Total internal reflection fluorescence (TIRF)^4^ microscopy (10) revealed eGFP-tagged M10 molecules move in a highly directed manner within the filopodia toward the tip region at a velocity of ∼0.9 μm·s^−1^ indicating that intrafilopodial trafficking is an active process.

**FIGURE 1.**
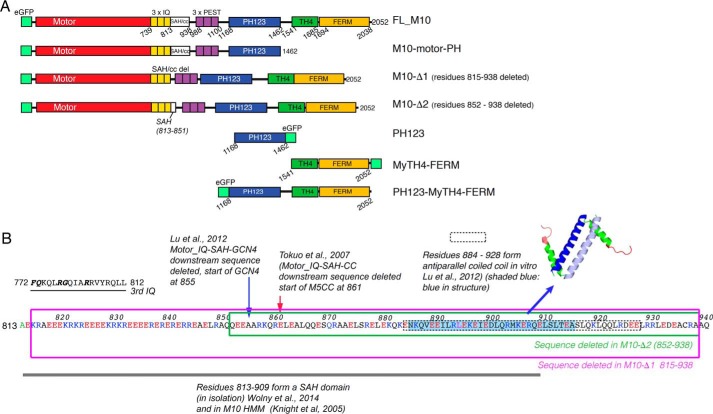
**Myosin 10 constructs.** *A,* diagram showing each of the constructs used in experiments to determine how different domains contribute to M10 behavior in cells. *Numbers* shown below each diagram refer to amino acid residue numbers. *B* shows the sequence of the SAH domain and anti-parallel coiled-coil domain, indicating which regions are deleted in M10-Δ1 and M10-Δ2. Residues 813–909 have been shown to form an SAH domain *in vitro*, whereas residues 885–913 have been shown to form an anti-parallel CC *in vitro*. In M10-Δ1, the entire SAH/CC sequence (815–938) is deleted. In M10-Δ2, only the last 86 residues (852–938) from this region are deleted. This deletes the CC and the distal part of the SAH domain, leaving the EKR-rich region of the SAH domain intact. Also indicated are the truncations investigated in two earlier cell biological studies, where the distal part of the SAH domain and the anti-parallel coiled-coil region were both deleted and replaced with either the coiled-coil forming region from myosin 5a (M5CC) or a GCN4 zipper motif.

Like other myosins, M10 uses the free energy of ATP hydrolysis to produce directed movement along actin filaments (3). Its structure consists of a canonical N-terminal motor “head” that binds actin and catalyzes the hydrolysis of ATP, a neck region comprising three IQ motifs followed by an extended sequence (125 amino acids) that is known to form an α-helical structure (11, 12), and finally, a C-terminal “tail” composed of a PEST domain, three pleckstrin homology (PH) domains, a myosin-tail-homology-4 (MyTH4), and a four-point-one ezrin-radixin-moesin (FERM) domain (Fig. 1, *A* and *B*) (5).

Although class 2, 5, and 18a myosins are known to dimerize via an α-helical coiled-coil structure within their tail regions (13, 14), the dimerization state of M10 remains controversial. It was originally assumed to dimerize via a 125-amino acid-long α-helical region (5), but subsequent experiments show that much of this sequence instead forms a stable α-helix (SAH) that does not dimerize (11, 15, 16), and isolated full-length M10 was shown by electron microscopy to be mainly monomeric (17). However, a recent structural study (18) has revealed that the C-terminal 53 amino acids of the α-helical region form a weakly paired anti-parallel coiled-coil (*K_d_* = 0.6 μm), which may dimerize when the local M10 concentration is high (*i.e.* within the filopodium). Weak dimerization has been reported for other myosin families (*e.g.* myosin 6) where it has been suggested to play a role in regulating motor activity (19, 20). Dimerization is functionally important because when two motor heads are linked together they are then able to move in a hand-over-hand fashion and move processively along actin. Recently, single molecule mechanical and optical studies of actin-based motor activity of a heavy meromyosin, HMM-like, construct of M10 have shown that it produces a power stroke of ∼17 nm (21), and at low loads it functions as a processive motor (21–23). However, for technical reasons, all of these studies were performed using an artificially dimerized recombinant form of M10, with either a C-terminal leucine zipper motif appended after residues 920 (24) or 936 (21) or the coiled-coil forming region of myosin 5a appended after residue 979 (23). A recent study has clarified the structural consequences of making such sequence alterations around the region of the SAH domain and coiled-coil forming motif (25). We can conclude from these studies that when M10 dimerizes via its anti-parallel coiled-coil forming region, its ability to move processively along the fascin-bundled actin core makes it well suited to cargo transportation toward the tip of the filopodium.

Given that an individual mammalian cell can express more than 12 different myosin isoforms (26), it is not clear how they are targeted to different regions of the cell or how their different activities are coordinated and regulated. It is likely that the modular domain structure of the myosin tail plays an important role in directing it to different cargos and also different locations within the cell. An interaction between the M10 motor domain and its globular tail has previously been shown to inactivate the ATPase (17). The same mechanism was also found for the closely related myosin 7a, which also has an MyTH4-FERM tail domain (27). It seems likely that M10 is targeted to the plasma membrane, at least in part, through binding of its centrally located PH domains to phosphatidylinositol (3,4,5)-trisphosphate (PtdIns(3,4,5)P_3_) lipids (17, 28–31). Coincidentally, binding of PtdIns(3,4,5)P_3_ at PH1 and PH2 was found to release the inhibitory effect of the tail on the ATPase activity of the motor, and cross-linking studies showed that lipid binding causes M10 dimerization (17, 28). The MyTH4 domain also interacts with microtubules and with the C-terminal FERM domain (32–34).

M10's distinct localization to the filopodial tip and the fact that it has constitutive motor activity makes it a model system for understanding mechanisms of active protein translocation and targeting. The predominantly polarized organization of actin within cells (35) in principle provides a directional signal, tending to send plus-end directed myosin motors toward the cell periphery. However, the slow movement and limited processivity of M10 (21) means that net transport by motorized movement along actin would be slow and inefficient. By analogy with our biophysical understandings of how DNA-interacting proteins find specific binding sites on genomic DNA via a combination of three- and one-dimensional diffusion (36), we hypothesized that M10 might use a similar mechanism, but with increased dimensionality, to rapidly navigate the cytosol and plasma membrane and finally travel to the tip of the filopodium. To test this idea, we have used live-cell single molecule imaging to characterize the behavior of full-length M10 and a panel of truncated expression constructs, fused to eGFP (Fig. 1). Single particle tracking and autocorrelation analysis methods were used to quantify motion of individual molecules at different regions of the cell. The results provide new insights into how M10 moves within the cell body and localizes to the filopodial tip.

## Results

### 

#### 

##### Full-length Myosin 10 Localizes to Filopodial Tips and Binds Intermittently at the Plasma Membrane

**FIGURE 2.**
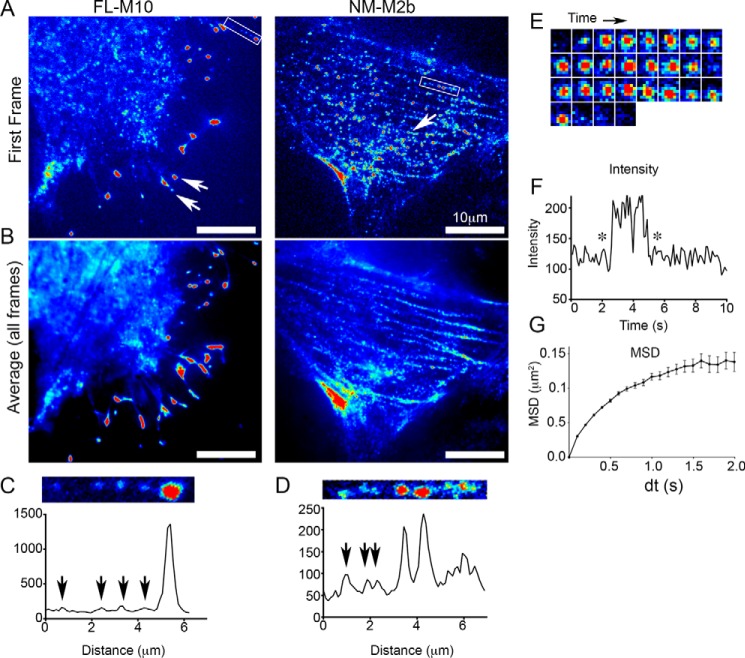
**TIRF images of FL-M10 and NM-M2b and analysis of intensities and FL-M10 spot mobilities.** *A,* single video frames (50-ms exposure) are shown for FL-M10 and NM-M2b transiently expressed in HeLa cells. *Arrows* indicate fluorescent spots, which are concentrated at the tips of filopodia for FL-M10 or on stress fibers in central regions of the cell for NM-M2b. *B,* averaged images obtained from 50 consecutive video frames from the same recordings shown in *A* (equivalent to 2.5-s total exposure) for FL-M10 and NM-M2b. FL-M10 does not localize to stress fibers, in marked contrast to NM-M2b. *C,* fluorescence intensity profile of FL-M10 measured along the length of a single filopodium (*boxed* in *A*). *Arrows* indicate fluorescent spots with intensities characteristic of a single fluorophore. *D,* fluorescence intensity profile of NM-M2b for a region along a single stress fiber (*boxed* in *A*) for NM-M2b. *Arrows* indicate that fluorescent spots are more variable in intensity than those seen for FL-M10 (shown in *C*). *E,* sequence of images shows intensity fluctuations of an individual fluorescent spot in a series of video frames originally captured at 20 frames/s (50-ms exposure). The highly magnified, 1 × 1 μm^2^, region located at the center of the FL-M10-expressing cell (shown in *A* and *B*) shows a fluorescent spot arriving at the basal plasma membrane, remaining bound at the membrane for several frames, before either unbinding or bleaching within a single video frame (the displayed images are 100 ms apart in time). *F,* intensity changes of the spot shown in *E* are plotted as a function of time. *Asterisks* indicate the initial and final frames shown in *E. G,* example of the MSD plotted against time interval (d*t*) for all fluorescent objects that were tracked from the record shown for FL-M10 in *A*.

Live cells, which had been transiently transfected with full-length M10 fused to eGFP (termed FL-M10), were visualized by TIRF microscopy. Consistent with earlier studies (4), the cells produced numerous filopodia, with FL-M10 localized at their tips (Fig. 2, *A* and *B, left panels*). Video imaging revealed individual fluorescent objects of diffraction-limited size and intensity similar to a single eGFP, which moved rapidly at the basal plasma membrane and trafficked within the filopodia (Fig. 2, *A* and *C*). In addition to filopodial and plasma membrane localization, there was also a diffuse background fluorescence signal within the cell body arising from fluorescent protein present within the cytosol, but not bound to the plasma membrane (Fig. 2, *A* and *B*). The absence of localization of FL-M10 at stress fibers or other actin-rich regions of the cells is in marked contrast to cells transfected with non-muscle myosin 2b fused to eGFP (NM-M2b) as demonstrated by single-frame and time-averaged TIRF images (Fig. 2, *A* and *B, right panels*). NM-M2b appeared as bright fluorescent puncta with a wide range of individual intensities that localized along the stress fibers (Fig. 2*D*) and did not localize to filopodia.

In addition to the FL-M10 clusters at the filopodial tips, FL-M10 molecules showed a number of behaviors as they moved at other regions of the cell. To analyze the patterns of movement, individual fluorescent objects of diffraction-limited size and intensity similar to that of a single eGFP were identified and tracked by computer (37). Fluorescent objects were most readily identified when they bound and then moved at the plasma membrane. They could then be tracked for periods of several seconds (Fig. 2*E*) before they disappeared within a single video frame (*i.e.* ∼50 ms) due to either photobleaching or detachment from the membrane (Fig. 2*F*). The spatial trajectories of the objects were analyzed by plotting the mean squared displacement (MSD) *versus* time interval (d*t*). These plots (Fig. 2*G*) were curvilinear (concave downwards) for the overwhelming majority of objects. Such behavior is inconsistent with a simple Brownian random walk, which would give rise to a linear straight-line graph. Only very few objects exhibited MSD *versus* d*t* plots with either linear or concave upward appearance typical of unrestricted random walk or motorized and persistent motion in a preferred direction (respectively). When such events are rare, as in our data sets, it is difficult to distinguish between instances of true motorized motion and random statistical variation, because short duration Brownian walk trajectories will sometimes appear to be highly directed, just by chance.

**FIGURE 3.**
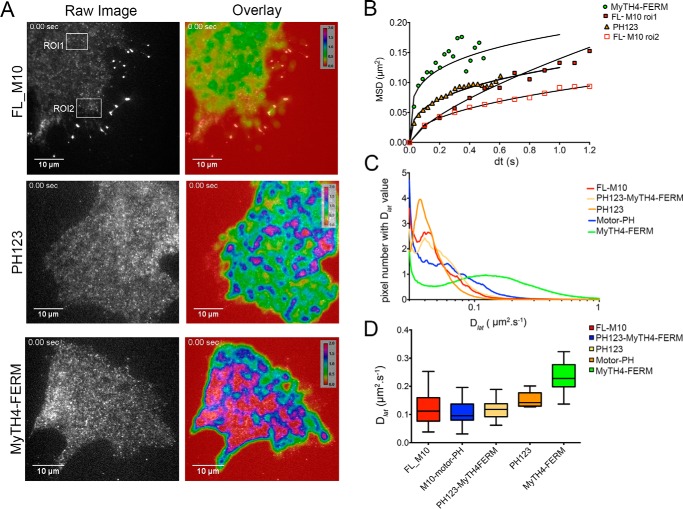
**Single particle tracking and autocorrelation analysis.** *A,* images show a single frame from the record (*Raw Image*) for three different constructs. A false-color scale (from low mobility (*red* = 0 μm^2^·s^−1^) to high mobility (*magenta/crimson* > 2 μm^2^·s^−1^) has been used to indicate the range of mobilities of the molecules analyzed in the accompanying image (*Overlay*). Regions of relatively low mobility (*yellow*) are observed at the plasma membrane of each cell. The MyTH4-FERM construct shows the largest area of highly mobile molecules. *B,* positions of individual fluorescent spots were tracked over time, and their MSD was plotted as a function of time interval (d*t*). Data are shown for different constructs and also for FL-M10 tracked at regions of interest (*ROI*) either close to the cell body (*ROI-1, upper boxed area* in *A*) or the cell periphery (*ROI-2, lower boxed* area in *A*). *C,* autocorrelation analysis shows the distribution of mobilities of spots over the cell surface (see also the mobility maps that are overlaid on *right-hand panels* of *A. D,* initial gradient of MSD *versus* d*t* plots for each particle track was used to estimate *D*_lat_. The distribution of *D*_lat_ values for each expression construct are shown as “box and whisker” plots (the “*boxes*” shows interquartile range; *cross-bar* is the median, and “*whiskers*” show maximum and minimum values). Stationary objects, where mobility is <0.02 μm^2^·s^−1^, were excluded from the analysis. All analysis was performed on transiently transfected HeLa cells at 37 °C.

The main conclusion from these results is that the overwhelming majority of FL-M10 molecules diffuse at the plasma membrane in an anomalous fashion, moving less far than expected at longer observation times (Fig. 2*G*). The short-range lateral diffusion coefficient (*D*_lat_) determined from the initial gradient of MSD *versus* d*t* plots was 0.2 μm·s^−1^, which is similar to other membrane-associated proteins (38, 39). We noticed that the anomalous diffusive behavior was more pronounced at the cell periphery than at the center of the cell (*i.e.* compare MSD *versus* d*t* plots obtained for two different regions of interest shown in Fig. 3, *A, top left,* and *B* (*red open versus red-filled squares*)).

In addition to the fluorescent objects that diffused relatively slowly at the plasma membrane and could therefore be identified and tracked by computer, visual inspection of the video recordings revealed a fluctuating and speckled fluorescent background signal arising from the rapid motion of cytosolic molecules diffusing within the evanescent field. To map the average rate of diffusion of all fluorescent objects at a particular location in the cell, we performed temporal autocorrelation analysis on the video data. The locally averaged autocorrelation time, τ, of the intensity fluctuations measured at each pixel location was calculated, and the value was converted to a pseudo-color map that reports the average rate of diffusion of all objects moving at a given pixel location. The maps were superimposed over the original fluorescence video data (see Fig. 3*A*, *right panels*, supplemental Movie 1, and “Experimental Procedures” for further technical details).

This analysis shows that the local diffusion coefficient is highest around the cell center and lower at the cell periphery. The maps also revealed “hot spots” within the cell, where molecules exhibited rapid diffusion (short correlation time). These regions correspond to areas that contain mainly cytosolic molecules and relatively few membrane-bound molecules. The rapid movement of FL-M10, particularly at the central regions of the cell, demonstrates that a significant fraction of the molecules are cytosolic and do not associate strongly either with the actin cytoskeleton or plasma membrane.

##### Intermittent Binding Behavior of Isolated M10 Tail Domains at the Cell Membrane

To understand how the tail domains of M10 act to target the full-length molecule to different regions of the cell, a panel of eGFP-tagged recombinant proteins in which the motor region had been deleted were tested (*i.e.* the lower three constructs shown in Fig. 1*A*). As for FL-M10 (above), the different protein constructs were transiently transfected into live cells that were then viewed by TIRF microscopy. The movement of molecules that bound at the plasma membrane was again assessed by single particle tracking and analysis of MSD *versus* d*t* plots (Fig. 3*B*).

Individual MSD *versus* d*t* plots were curvilinear (concave downwards) for all of the constructs, as found for FL-M10 (Fig. 2*G*). All constructs containing PH domains had short-range *D*_lat_ values that were similar to FL-M10 (Fig. 3, *B–D*). However, the isolated MyTH4-FERM construct behaved differently and exhibited higher mobility than the other constructs tested (Fig. 3*B*, *green circles*). This finding was confirmed by autocorrelation analysis, which also showed that the MyTH4-FERM construct had a higher mobility than either PH123 or FL-M10 (Fig. 3, *C* and *D*, and supplemental Movies 2–4). The construct containing both MyTH4-FERM and PH123 (PH123-MyTH4FERM; Fig. 1*A*) had similar mobility to PH123 and FL-M10 indicating that the low mobility conferred by PH domain binding at the plasma membrane dominated the behavior of FL-M10. The low plasma membrane affinity and high mobility of MyTH4-FERM suggested that this region of the molecule is less important for targeting FL-M10 to the plasma membrane.

##### Filopodial Trafficking of FL-M10

**FIGURE 4.**
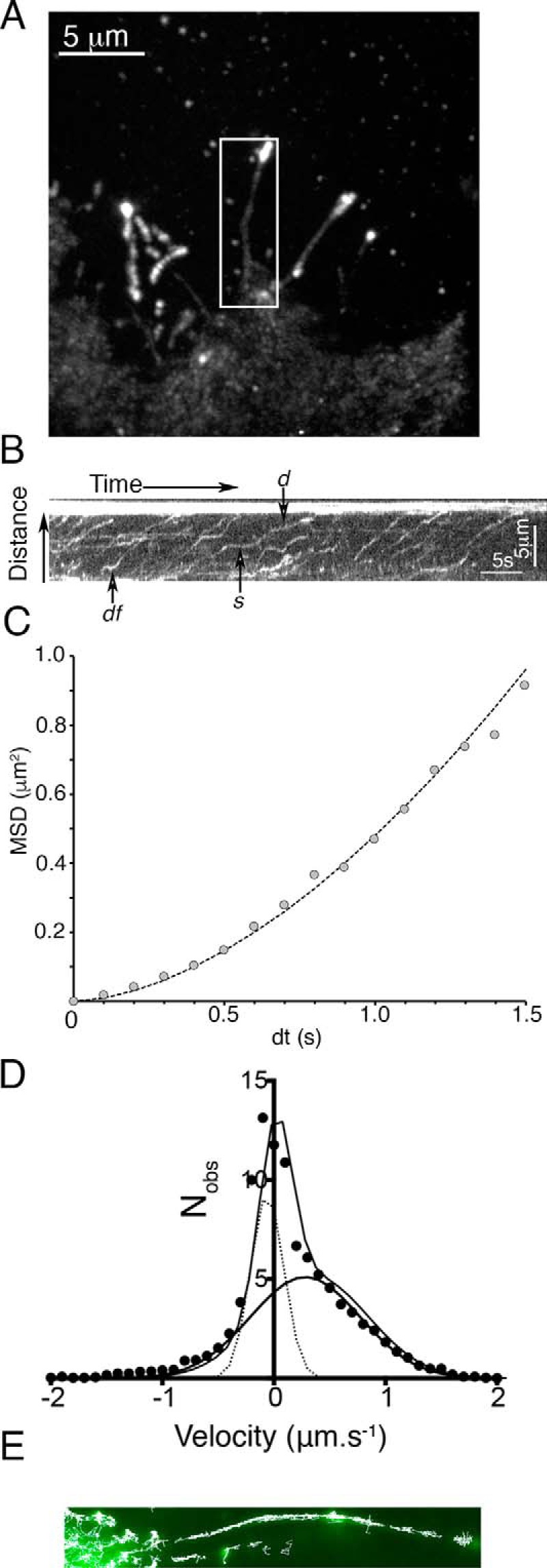
**Analysis of the behavior of FL-M10 in filopodia of HeLa cells.** *A,* TIRF image (average of 50 video frames, equivalent to a 2.5-s exposure) of a HeLa cell expressing FL-M10 with a *boxed region* around a single filopodium. The filopodial backbone was manually traced and fitted to a spline so that a 5-pixel wide stripe along the path of the filopodium could be “straightened” (using ImageJ) and a kymograph generated. *B,* kymograph obtained from the boxed filopodium's (shown in *A*) fluorescence intensity along the length of the filopodium plotted on the *ordinate* is shown as a function of time (along the *abscissa*). The bright spot at the tip of the filopodium is at the *top* of the figure. *Arrows* indicate the following: *d,* directed movement; *s,* stalled movement; *df,* diffusive movement (see supplemental Movie 1). *C,* single particle tracking allowed the motion of individual particles to be quantified, and most (>80%) exhibited periods of smooth motion directed toward the tip, typified by a quadratic relation between MSD and d*t. D,* however, when the instantaneous velocities were histogrammed, more complex behavior became apparent. The distribution of particle velocities required a fit to two Gaussian functions. One type of motion had an average instantaneous velocity close to zero (*i.e.* stalled and diffusive behaviors), and the other had an average positive velocity, indicating directional (motorized) motion toward the filopodial tip. *E,* particle trajectories were determined with sub-pixel resolution and plotted on top of one another to create a composite image showing the accumulated individual tracks (*white lines*) overlaid onto the averaged fluorescence image of the filopodium. The individual tracks cluster along a central path, ∼100 nm in diameter, consistent with EM images of the filopodial actin core (this observation is explored further in Fig. 5).

Analysis of FL-M10 movement within filopodia (*boxed region*, Fig. 4*A*) using kymographs (supplemental Movie 1) showed a mixture of persistent tip-wise motion, stalling and random back-and-forth motions within the filopodia (Fig. 4*B*). The velocity of molecules while undergoing smooth directed movement was ∼1.4 μm·s^−1^, similar to that measured previously for M10 *in vitro* at 37 °C (21) and in live cells (10, 40). Single particle tracking of individual spots within the filopodium revealed that ∼80% of the MSD *versus* d*t* plots showed a concave upward relationship, characteristic of motorized movement directed toward the filopodial tip (Fig. 4*C*). However, when data for all objects were pooled, it was apparent that the trajectories did not show continuous directed motion but nearly all exhibited periods of diffusive motion and stalled behavior. In earlier studies, the characteristic run length of myosin 10 was determined by histogramming the duration and distance moved by individual eGFP-tagged molecules along surface-immobilized actin filaments or filament bundles (23, 24). In such studies, the start and end of each event is relatively easy to score, because the fluorescence signal shows abrupt changes in intensity as molecules first arrive at a surface-bound actin filament and finally unbind and diffuse away from actin when the run terminates. In this study, run-length is more difficult to estimate because myosin 10 is confined within the filopodium so the fluorescent signal remains essentially constant until the eGFP photo-bleaches. Visual inspection of kymographs presented here and in earlier studies (10, 40) reveals many molecules moving in a seemingly continuous fashion for distances of up to 5 μm implying that the mean run-length is higher than *in vitro* estimates. If mean run-length were ∼1 μm (23, 24), then continuous translocation >5 μm should occur for less than 1% of observations. However, the results are not incompatible because myosin 10 molecules moving within the filopodia are essentially trapped between the fascin-bundled actin core, and the bounding plasma membrane and the long distance motion observed in the kymographs can be explained if myosin 10 briefly detaches from actin but then rapidly rebinds and continues moving.

To explore the behavior of individual molecules within the filopodium, we calculated instantaneous velocities (measured over a moving 500-ms time window) for all objects that had been tracked and histogrammed the distribution of velocities (Fig. 4*D*). The histograms had a peak value centered around zero, with a shoulder on the right-hand side (*i.e.* positive velocities directed toward the filopodial tip). The histograms were then fitted to the sum of two Gaussian distributions. One population had a net velocity close to zero (−0.06 ± 0.21 μm·s^−1^, mean ± S.D.); corresponding to periods of either stalled and random back-and-forth motion. The other population had an instantaneous velocity of +0.28 ± 0.69 μm·s^−1^ (Table 1), corresponding to periods of movement directed toward the filopodial tip. Note that the mean velocity estimates obtained from the histograms are lower than those measured for periods of smooth tip-wise movement measured from the kymographs (see above).

**TABLE 1:** **Instantaneous velocities for eGFP and M10 constructs in filopodia of transiently transfected HeLa cells** V1 and V2 are the mean velocity values derived from velocity distribution histograms (shown in Fig. 6*E*) that were fitted to the sum of two Gaussian functions. *N*(cells) and *N*(obj) are the number of cells and the number of tracked objects (respectively), and “*n*” is the number of objects assigned to the “V2” population. All data obtained at 37 °C except *eGFP, which was at 25 °C.

Construct	*N* (cells)	*N* (obj)	V1 ± S.D.	V2 ± S.D. (*n*)	%V2
			μ*m*·*s*^−*1*^	μ*m*·*s*^−*1*^	
*eGFP	5	378	−0.06 ± 0.01	N/A	0%
FL-M10	23	425	−0.06 ± 0.21	0.28 ± 0.69 (149)	35%
M10-motor-PH	12	374	−0.02 ± 0.31	0.33 ± 1.18 (112)	30%
M10-Δ2	4	159	−0.06 ± 0.19	0.23 ± 0.78 (43)	27%
M10-Δ1	19	275	−0.04 ± 0.18	0.15 ± 0.60 (124)	45%

**FIGURE 5.**
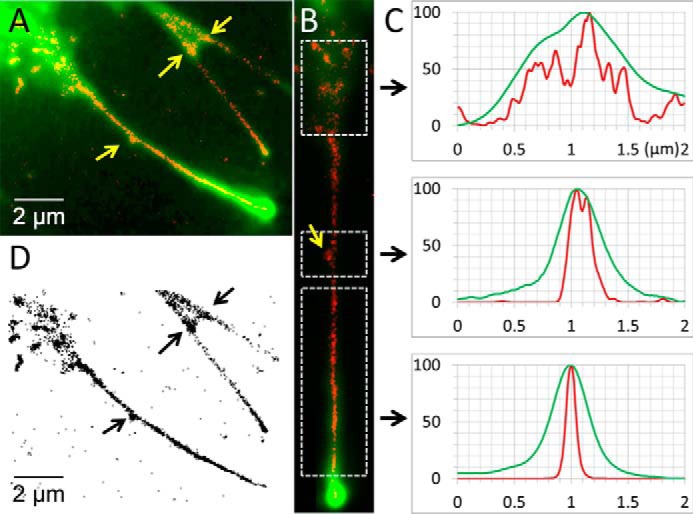
**Super-resolution image of the filopodium.** By accumulating all of fluorescent object localizations obtained during a video recording, super-resolution images of the paths taken by FL-M10 molecules within the live HeLa cell could be determined. *A,* example image (showing three adjacent filopodia) illustrates how paths taken by FL-M10 become channeled into the main filopodial shaft. Individual spot localizations (*red*) are superimposed over the average fluorescence data (*green*). The example chosen here shows interesting ultrastructural features that were observed in about 10% of filopodia (indicated by *yellow arrows*); there is a bulge on the side of one filopodium and a connecting bridge between two filopodia on the *upper right. B,* longer filopodium, shown in *A*, was computationally straightened, and the intensity profile across the breadth of the filopodium (averaged over the length of the *boxed regions*) was plotted in the corresponding panels in *C*. The diameter of the filopodium estimated from analysis of the average fluorescence image (shown in *green*) and the estimate obtained by the individual FL-M10 spot localizations (*red*) show that although the filopodial diameter estimated toward the base is similar by both methods, it starts to diverge toward the mid-shaft region of the filopodium. Super-resolution data shows that FL-M10 molecules are confined within an average filopodial diameter of 116.5 ± 18 nm (full-width at half-maximum height), whereas the diffraction-limited average fluorescence image reports 357 ± 28 nm (*n* = 39). Note that the *central panel* corresponding to the region containing the sub-diffraction limit, ultrastructural, feature within the filopodial shaft confirms that the super-resolution data (in *red*) exhibits two peaks separated by ∼100 nm, whereas the original fluorescence data (in *green*) shows a single, but slightly broadened, peak. *D,* super-resolution particle localizations are plotted on an inverted grayscale so that the sub-diffraction limit ultrastructural features are easier to visualize.

Because the single particle tracking analysis was performed with sub-pixel (∼30 nm) resolution, the paths taken by individual molecules can be plotted over the original fluorescence image to reveal structural features that are smaller than the diffraction limit (Fig. 4*E*). These plots indicate that the individual paths cluster along a central region, ∼100 nm in diameter, consistent with electron micrographs of the filopodial actin core structure (41). This observation was explored further (Fig. 5) by plotting all of the single object centroid localizations, accumulated over an entire video recording (from 10 to 100 s duration), over a computationally expanded copy of the averaged image data. Linear interpolation was used to create a 16-fold increase in image size, leading to a reduction in pixel size (from 100 to ∼6.3 nm). Each single object localization was summed onto the image canvas as a Gaussian blurred spot with centroid amplitude proportional to original spot intensity and spread proportional to the inverse square-root of intensity (using a red scale look-up table) (Fig. 5*A*). Such super-resolution images revealed interesting structural features including small defects in the filopodial shaft structure and thin bridging structures, which link adjacent filopodia. The super-resolution reconstructions show that filopodia project from a root region (∼1 μm across) that rapidly tapers to a shaft diameter of around 116 nm (Fig. 5, *B* and *C*). Small imperfections (defects) in filopodial ultrastructure were present in about 10% of filopodia. Although movement of FL-M10 in the cell body was chaotic at distances greater than 2–3 μm from the filopodial base (as discussed before), we observed directed motion in the “root region” of filopodia on many occasions (see supplemental Movie 5, which should be played as a “looped” movie).

##### Role of MyTH4-FERM Domain

**FIGURE 6.**
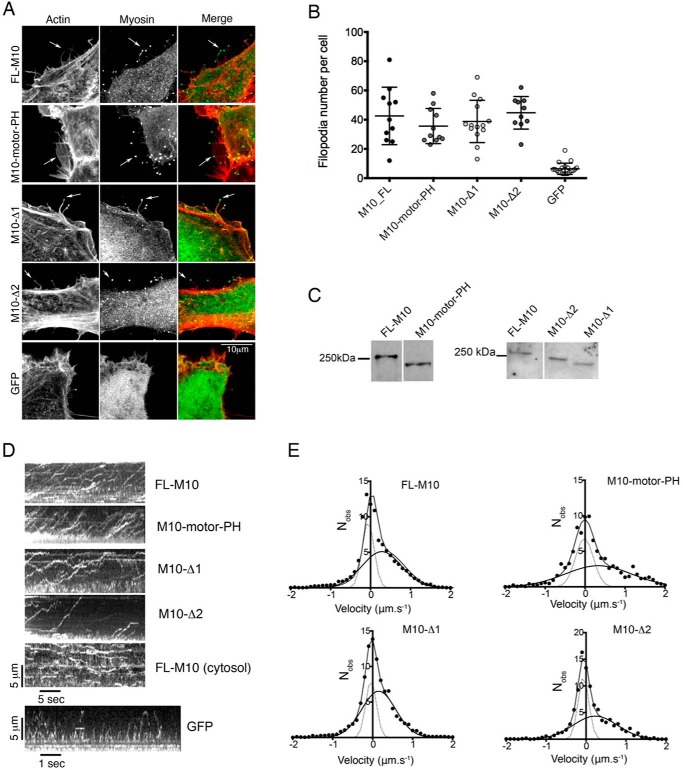
**Effects of M10 deletion constructs on filopodia number and velocity.** In these experiments, M10 expression constructs in which either the C-terminal MyTH4-FERM domain (M10-motor PH) or the entire SAH/CC domain (M10-Δ1) were deleted or the ERK-rich region of the SAH (residues 815–851) were left intact and the remainder of the SAH domain/cc was deleted (M10-Δ2) were expressed in HeLa cells. *A,* all constructs accumulate at the tips of filopodia, as found for FL-M10. *B,* number of filopodia per cell, for each of the different constructs and for eGFP-expressing cells, are plotted with *bars* indicating mean and S.D. Filopodia number was counted for a minimum of 10 cells. The numbers of filopodia for each of the constructs is significantly higher (*p* < 0.01) than the number of filopodia in cells transfected with eGFP alone. *C,* Western blots of HeLa cells transfected with the FL-M10, M10-Δ1, M10-Δ2, and M10-motor-PH constructs. Bands detected with anti-eGFP antibody. *D,* top four kymographs show the trafficking of the different M10 expression constructs within filopodia (data for FL-M10 repeated from Fig. 4*B*). A kymograph was also constructed for a representative central region of a cell expressing FL-M10. The bottom kymograph, for GFP (eGFP) alone, moving within a filopodium, exhibits no fluorescence accumulation at the filopodial tip and shows rapid back-and-forth motion (note the faster time scale for this kymograph). *E,* distribution of immediate particle velocities determined from single particle tracking analysis over a running window of 10 video frames (500 ms time window) were determined for each construct (FL-M10, M10-motor-PH, M10-Δ1 and M10-Δ2) and histogrammed. The histograms were then fitted to the sum of two Gaussian functions.

To test whether the MyTH4-FERM domain was required for targeting FL-M10 to the plasma membrane and/or the filopodial tips, we used an expression construct in which the C-terminal MyTH4-FERM domain was deleted (M10-motor-PH, Figs. 1*A* and 6*C*). Cells transfected with M10-motor-PH produced the same number of substrate-adherent filopodia as cells transfected with FL-M10, and there was clear localization of M10-motor-PH to the filopodial tips (Fig. 6, *A* and *B*). Furthermore, the mobility and fraction of plasma membrane-bound M10-motor-PH was also indistinguishable from FL-M10 (Fig. 3, *C* and *D*). Kymographs of M10-motor-PH trafficking were similar to FL-M10 (Fig. 6*D*), and the velocity measured from the kymographs was again ∼1.4 μm·s^−1^ as measured for FL-M10. Single molecule tracking additionally showed that the filopodial trafficking for this construct was also similar to FL-M10. The instantaneous velocity distribution analysis again indicated two populations (Fig. 6*E*), one with a velocity close to zero (Table 1) and the second with an instantaneous velocity that was not significantly different from M10-FL (Table 1).

##### Deletion of the Coiled-coil and SAH Domains Slows but Does Not Abrogate Filopodial Trafficking

We next determined whether deleting the SAH domain from the FL-M10 construct affected the behavior of M10. Residues 809–932 were originally predicted to form a coiled-coil (5). A peptide containing the first 36 residues was subsequently found to form an SAH domain in isolation, whereas a longer sequence (∼100 residues) was found to form an SAH domain by measuring rotary shadowed images of M10-HMM (11). More recent results showed that a peptide containing residues 813–909 form an SAH domain in isolation (12), whereas a peptide containing residues 884–934 form an anti-parallel coiled-coil (“CC”) (18). Therefore, we made two expression constructs as follows: one in which the entire SAH/CC sequence is deleted (Δ815–938), termed M10-Δ1 (Fig. 1*B*); and the other had only the last 86 residues deleted (Δ852–938), removing just the distal part of the SAH domain and the entire CC sequence, termed M10-Δ2.

Analysis of cells transfected with M10-Δ1 and M10-Δ2 expression constructs (Fig. 6) showed that filopodial formation and tip localization were unaffected by deletions encompassing the SAH and coiled-coil forming regions (residues 815–934). There was no significant difference in number of filopodia (Fig. 6*B*) for cells transfected with M10-Δ1 or M10-Δ2 compared with cells transfected with either FL-M10 or M10-motor-PH constructs. Kymograph analysis of filopodial trafficking for M10-Δ1 and M10-Δ2 also gave similar results to the FL-M10 and M10-motor-PH constructs, and the velocity measured directly from the kymographs was 1.4 μm·s^−1^ (Fig. 6*D*). Intrafilopodial trafficking again indicated two types of behavior (Fig. 6*E*). The major population had zero net velocity (freely diffusing and stalled molecules), and the other showed directed movement toward the filopodial tip (Table 1). The mean velocity of the moving fraction of M10-Δ1 was 0.15 ± 0.6 μm·s^−1^, which was significantly slower (∼50%) than FL-M10 (one-tailed *t* test, with sample size, *n*, reduced in proportion to the moving fraction, gave *p* < 0.05). However, the mean velocity of the M10-Δ2 moving fraction was increased close to (and not significantly different from) FL-M10 (0.23 μm·s^−1^, Fig. 6, *D* and *E,* and Table 1). The difference between the two mutant proteins, M10-Δ2 *versus* M10-Δ1, was not statistically significant (*p* > 0.05).

## Discussion

We have used TIRF-based single molecule imaging to track the fate of individual fluorescently tagged molecules within live mammalian cells to help elucidate how M10 is targeted to the tips of filopodia. Simple visual inspection of the TIRF imaging video data revealed that full-length M10 shows three distinct types of behavior as follows: it diffuses freely within the cytosol, moves more slowly when bound to the plasma membrane, and then in a directed manner within the filopodia. We quantified these observations using a combination of single particle tracking, kymographs, and correlation analysis. By studying different deletion mutants, we have dissected which regions of the M10 molecule are important for each type of behavior. Our study leads to a model for M10 trafficking in which diffusive and active movements combine to rapidly target myosin 10 to the filopodial tip.

Our data show that M10 moves rapidly through the three-dimensional volume of the cell by diffusion (where the diffusion coefficient, *D*_lat_, > 5 μm^2^·s^−1^), and periodically explores regions of plasma membrane by performing intermittent two-dimensional diffusional movements, with a lateral diffusion coefficient at the membrane, *D*_lat_ ∼0.2 μm^2^·s^−1^. Then, after locating an appropriate region of membrane at the base of the filopodium, the myosin motor is activated, and the molecule moves in a highly directed manner along the polarized actin filament bundle within the filopodium at a maximum velocity of ∼1.4 μm·s^−1^. Once at the tip, the M10 molecules are essentially trapped and therefore accumulate as a characteristic cluster or punctum. Even within the filopodium, M10 movement shows discontinuities in which smooth motion is interrupted by periods of stalled and diffusive motion, such that the averaged velocity over a 500-ms time window is much slower (∼0.3 μm·s^−1^) than for regions of smooth continuous motion seen on the kymographs (∼1.4 μm·s^−1^).

Directed movement does not require the C-terminal MyTH4 FERM domain (Table 1). Furthermore, although removal of the entire SAH domain and coiled-coil forming region (M10-Δ1) reduces the velocity of M10 trafficking, it does not affect filopodial number or tip localization, which implies that the SAH domain is not essential for initiation of filopodia. Our observation that filopodial number is similar to FL-M10 even when the speed of intrafilopodial trafficking is reduced implies the speed of M10 trafficking does not limit the rate at which filopodia are formed. However, the N-terminal region of the SAH domain (11) is important for full-speed movement of M10 within filopodia.

Previous studies have shown that deleting the distal SAH domain and CC region (Fig. 1*B*) and replacing these with a parallel coiled-coil did affect filopodia formation. However, none of these studies used constructs that included the tail domains, as we have done here, but only used truncated “HMM-like” constructs. In one study, an M10-HMM-like construct was artificially dimerized using the coiled-coil forming region of myosin 5 or by fusion with the inducible dimerization domain, FKBP, placed at residue 861 (Fig. 1*B*). This retains the EKR-rich region of the SAH domain but removes the distal region, as well as the anti-parallel CC sequence (42) and all of the downstream tail domain sequence. This construct did promote some filopodial formation, but the filopodia were short and unstable in the absence of tail domains. A subsequent study (18) showed that an “HMM-like” construct in which GCN4 was introduced at residue 855 to dimerize the myosin just after the EKR-rich SAH region (Fig. 1*B*) again did not show strong filopodial localization. A longer construct, in which the tail domains were added after the GCN4 zipper, showed an increased filopodial localization compared with the HMM construct, suggesting that the presence of the tail domains does play a role in filopodial localization. However, neither construct promoted an increase in filopodial number. Neither of these studies investigated any effects on M10 movement within the filopodia.

A drawback of both of these studies is that a parallel coiled-coil is placed next to the EKR-rich region (∼first 40 residues of the SAH domain), deleting downstream SAH and anti-parallel coiled-coil region. In this case, we would expect the EKR-rich region to remain as an SAH domain, as we found this was the case when we tested a myosin 5-SAH chimera (16). The effects of forcibly dimerizing M10 using a parallel coiled-coil forming GCN4 motif placed just after the EKR-rich region may make these constructs incompatible with filopodial formation and/or trafficking, although a simple deletion of either the whole SAH domain and coiled-coil may still allow M10 to initiate and traffic to the ends of filopodia. We would not expect either of the deletion constructs we made to heterodimerize with endogenous M10 through the anti-parallel coiled-coil region, as it was absent from both constructs. Thus, our results suggest that the anti-parallel coiled-coil domain is not essential for M10 to induce formation of surface-adherent filopodia or to localize to filopodial tips consistent with earlier findings (17). Perhaps our most surprising result is that both M10-Δ1 and M10-Δ2 both exhibit smooth directed motion within the filopodium. However, deletion of both the anti-parallel coiled-coil and proximal SAH domain (M10-Δ1) reduces trafficking speed by ∼50%. It remains a possibility that our transiently transfected mutant proteins may associate with cargo that is being transported by endogenous (wild type) M10, and the reduced trafficking speed might arise because the mutant proteins hinder the activity of the endogenous motor molecules. The way to test this possible explanation would be to use a knock-out cell line or CRISPR/cas9-targeted genome editing.

We found that FL-M10 associates with the plasma membrane in the cell body, where it diffuses in an anomalous fashion with a maximal rate typical of other membrane-associated proteins (∼0.2 μm^2^·s^−1^), suggesting that M10 is not able to move processively on cortical actin filaments while associated with plasma membrane in central regions of the cell, also it neither binds nor moves on actin stress fibers or cortical actin structures. Truncated constructs containing various combinations of M10 tail regions showed similar behavior and mobility to the full-length molecule, except for the isolated MyTH4 FERM domain, which had increased mobility and lower affinity for plasma membrane. Deletion of the MyTH4-FERM domain from the parent molecule did not affect either mobility at the plasma membrane or speed of trafficking within the filopodia. Neither did it affect the number of surface adherent filopodia, suggesting that this domain is not required for the motile behavior of this myosin. This finding is consistent with earlier studies that also showed the MyTH4-FERM domain is not required for the induction of substrate-attached filopodia in COS-7 cells (17, 43).

These findings additionally suggest that the motility and more particularly the immobilization of M10 at the plasma membrane is driven by weak interactions between the motor domain of M10 and the actin cytoskeleton and by PH domains to PtdIns(3,4,5)P_3_. This type of behavior was observable both in the cell body and in the filopodia, suggesting that FL-M10 can interact with actin and PtdIns(3,4,5)P_3_ in both regions of the cell but that the motor is able to move processively only when in the immediate vicinity of the filopodium as suggested by early studies (22). The requirement of the PH domains for correct function and localization of M10 is supported by earlier findings, which suggested that this domain is important for regulating M10 activity (17). Isolated molecules of full-length M10 imaged by electron microscopy form a compact structure similar to that seen for the related myosin, myosin 7a (27, 44). Moreover, M10 becomes activated when it binds to PtdIns(3,4,5)P_3_ (and to some extent to PtdIns(4,5)P_2_). Although that study showed the MyTH4FERM domain also interacted with the motor domain and was additionally implicated in regulating its ATPase activity, it seems that the PH/PtdIns(3,4,5)P_3_ interaction is most important for controlling this regulatory pathway. It has also been suggested that the binding to PtdIns(3,4,5)P_3_ induces formation of dimers of M10 (17), but our experiments cannot unequivocally determine whether molecules moving within filopodia are dimeric or not. However, it is not surprising that both the PH domains and the motor domain are required to immobilize M10 at the plasma membrane, and it is likely that this then leads to filopodial formation.

**FIGURE 7.**
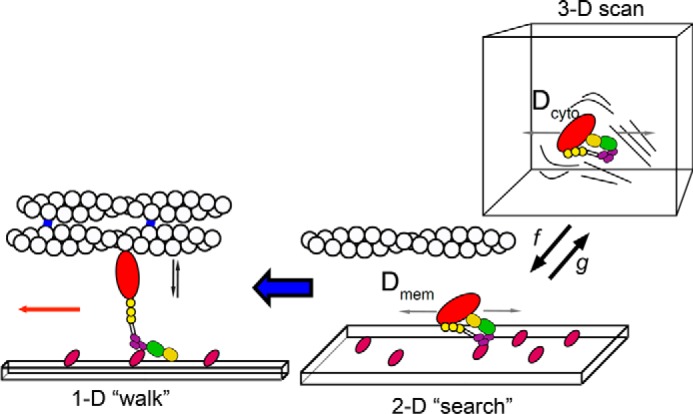
**How myosin 10 is targeted.** The schematic illustrates how M10 might move from the cell body out toward the cell periphery and eventually to the filopodial tip. In the cytosol, the motor is auto-inhibited and most likely monomeric, diffusing rapidly in the three-dimensional volume (*D*_cyto_ ∼5 μm^2^·s^−1^). M10 binds intermittently (with rate constants *f* and *g*) to phosphoinositide phospholipids at the plasma membrane and then undergoes two-dimensional diffusive motion at a rate typical of plasma membrane-associated proteins (*D*_mem_ ∼0.2 μm^2^·s^−1^). When the PH domains (*purple*) bind phospholipid, the motor is activated and may also associate with actin. The MyTH4-FERM domains (*yellow* and *green*) may additionally interact with their cargo proteins. When M10 localizes at the base of a filopodium, it can then move processively in a one-dimensional manner, along the fascin (*blue*) bundled actin filaments toward their barbed ends, which direct M10 molecules toward the filopodial tip.

M10 exhibits directed movement only at regions close to the base of the filopodium and shows clearly directed motion only after entering the main filopodial shaft that is composed of fascin-bundled actin. It has been suggested that the tropomyosin isoform TM3 (TmBr3/Tpm1.7), together with Arp2/3, helps promote filopodia formation by increasing the number of short filaments without capping proteins, and these then recruit the bundling protein fascin to generate filopodia (45). Certainly, tropomyosin isoforms are likely to help regulate the binding of different myosin isoforms to particular cellular regions (46). For example, Tpm3.1 has been shown to regulate the binding of non-muscle myosin 2A to cortical actin filaments (47), and its overexpression inhibits filopodia formation (45). Thus, the diffusive behavior of M10 in the cell body can best be described as a three-dimensional search for the correct lipid (PtdIns(3,4,5)P_3_) and then a two-dimensional search for the correct type of actin. Once it reaches the base of the filopodium (Fig. 7), it then start to move processively and perform a unidirectional one-dimensional walk along the filopodial actin bundle. Our single molecule imaging shows individual M10 molecules are often recruited at the base or root of the filopodium. The root region may act as a funnel where M10 is activated and recruited to the filopodium. An interesting consequence of this model is that the fastest way for an M10 molecule to search the plasma membrane and reach the base of a filopodium is to make the ratio of time that it spends diffusing in the cytosol *versus* time spent bound and slowly diffusing at the plasma membrane similar to the ratio of the two respective diffusion coefficients (*D*_cyto_ and *D*_mem_). Then, when a molecule binds at the plasma membrane it will explore a region given by Equation 1,

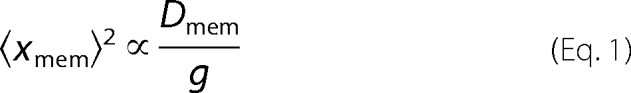
 and when it unbinds (with rate constant, *g*) and undergoes diffusion in the cytosol, it will move a similar distance as shown in Equation 2,

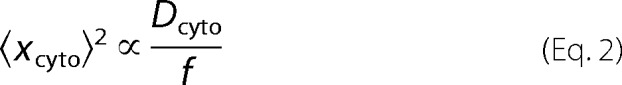
 before rebinding (with rate constant, *f*). This would allow a new region of the membrane to be explored each time the molecule rebinds. If we make a rough calculation using realistic values (*D*_mem_ = 0.2 μm^2^·s^−1^; *D*_cyto_ = 5 μm^2^·s^−1^; PH domain detachment rate *g* = 0.1 s^−1^ (48) and assuming a cytosolic concentration of 25 nm and diffusion-limited rebinding rate = 10^8^ m^−1^·s^−1^ giving *f* = 2.5 s^−1^), then for each mean dwell time of 10 s at the membrane and 0.4 s within the cytosol the M10 molecule would explore a region of ∼2 μm^2^. This may help to explain how M10 translocates rapidly and efficiently from the cytosol to the distant filopodial tips, which would presumably be much slower if targeting relied solely on random diffusion or on directed motion along the randomly oriented network of cytoskeletal actin filaments, stress fibers, and other cortical actin structures.

## Experimental Procedures

### 

#### 

##### Cloning of M10 Constructs

The full-length bovine myosin 10 cDNA (P79114) together with the tail constructs (PH123-MyTH4-FERM and MyTH4-FERM) were generous gifts from Prof. Richard Cheney (University of North Carolina) (4). The expression construct for non-muscle myosin 2b (NM-M2b) was a kind gift from Prof. Robert Adelstein (NHLBI, National Institutes of Health, Bethesda). In both constructs, eGFP is fused in-frame at the N terminus. eGFP-PH123 was reported earlier (48). A PCR-based approach was used to delete the SAH domain and anti-parallel coiled-coil (residues 815–938) to generate the construct termed M10-Δ1 (Fig. 1). Similarly, a second construct, M10-Δ2, was created in which part of the SAH domain, excluding the EKR-rich region, and the entire parallel coiled-coil region were deleted (residues 852–938). A truncated construct in which the MyTH4-FERM domains are missing was generated by introducing a stop codon after the PH123 domain but before the MyTH4 domain (at residue 1540) to generate M10-motor-PH. In each case, eGFP-Myo10 was used as a template. All constructs were sequenced to confirm sequence fidelity and sizes confirmed by Western blotting.

##### Cell Culture and Transient Transfection

The recombinant M10 constructs were transfected into HEK293 or HeLa cells (no differences were observed in results for these two cell types). Cells were plated onto glass coverslips and transfected using either FuGENE 6 (Roche Diagnostics, Burgess Hill, West Sussex, UK) or Gene-juice (Millipore Ltd., Watford, Herts, UK) following the recommended protocols. 12–16 h later, the cells were either fixed and then stained for actin using Alexa-546 phalloidin (ThermoFisher Scientific, Hemel Hempstead, HERTS, UK) or prepared for live-cell imaging.

##### Western Blotting

Cells were plated into a single well of a 6-well plate, before transfecting as described above. 24–48 h later, cells were washed with PBS and then scraped into Laemmli buffer. Samples were run on a 7.5% acrylamide gel, transferred to nitrocellulose, blocked in PBS containing 5% milk for 1 h, then incubated with an anti-GFP antibody (Abcam), washed in PBS/Tween, and then incubated with an anti-rabbit horseradish peroxidase antibody before exposing to film (Fig. 6*C*).

##### Live-cell Imaging

The cell growth medium was replaced with Hanks' balanced salt solution with 20 mm HEPES (pH 7.4) and 10% serum, and the coverslip was assembled into a small chamber for viewing the cells on an inverted microscope at 37 °C in a custom-built total internal reflection fluorescence microscopy system (see below). Approximately 10% of the cells had a suitable level of expression, such that single molecules could be visualized as single spots when viewed by TIRF microscopy. This equates to a final concentration of eGFP fusion protein in cells in the nanomolar range (*i.e.* ∼1 molecule per μm^3^; ∼5000 molecules/cell).

##### TIRF Microscopy

The TIRF imaging system has been described elsewhere (29, 38); however, we present a brief description as follows. The beam from a 100-milliwatt 556-nm laser (MGL-556-100, Suwtech, Shanghai, China) was expanded by a Galilean beam expander and focused at the back focal plane of a high numerical aperture objective lens (Alpha Plan, ×100, NA 1.45, Carl Zeiss Ltd., Cambridge, UK). A front-surface silvered mirror (3 mm diameter) was used to direct the laser beam into the objective lens by positioning it immediately below and at the extreme edge of the back aperture. The average laser intensity at the specimen plane was ∼40 microwatts ·μm^−2^. The incident laser beam angle was adjusted to 64° to create the evanescent field at the glass-water interface. A digital EMCCD camera (iXon897BV, Andor Technology Ltd., Belfast, UK) was used to acquire video sequences that were stored directly on a computer hard drive using a computer frame-grabber card and proprietary software. The microscope's image magnification was calibrated using a reticule, giving 100 nm per pixel in both *x* and *y* camera axes. Experiments were performed at 37 °C, and video records were collected at either 10, 33, or 50 frames·s^−1^. The fluorescence intensity of single GFP molecules was characterized using control specimens in which GFP was antibody immobilized at very low surface density (<1 fluorescent spot per 1 μm^2^) and imaged under identical conditions (*i.e.* same laser intensity, camera gain, exposure time, and so forth) to our live cell imaging experiments.

##### Data Analysis

Fluorescent spots that localized to the plasma membrane were automatically detected and tracked using custom-written image analysis software, GMimPro (37) and ImageJ (49). Molecules that bound at the plasma membrane diffused at a speed determined by lipid mobility (*D*_lat_ <0.2 μm^2^·s^−1^), which was sufficiently slow to allow the path of individual molecules to be tracked between adjacent video frames using a “nearest-neighbor” tracking algorithm (37). Furthermore, analysis of the single molecule trajectories was then performed using Excel (Microsoft, version 2010) or IgorPro (WaveMetrics Inc, Lake Oswego, OR). Trajectories of molecules, moving within filopodia, were also analyzed using kymographs generated by a simple ImageJ macro (available on request).

##### Autocorrelation Analysis

Fluorescent objects that moved freely within the cytosol were too fast to be individually tracked because their paths could not be unambiguously determined between adjacent video frames. Instead, we used autocorrelation analysis to estimate the speed of objects moving at each pixel location. The autocorrelation time, τ, depends upon the time it takes a molecule to traverse an optical volume, which is a convolution of the rate of diffusion, the imaging point spread function, evanescent field depth, and camera pixel size. Autocorrelation analysis was conducted at each pixel location, across the entire field of view (up to 512 × 512 pixels), for the duration of the video record. The characteristic autocorrelation time, τ, was then calculated at each pixel position. The τ values were then locally averaged over an 8 × 8 pixel window size (computations were performed using IgorPro). The resulting matrix of values was converted to diffusion coefficient values by performing identical analysis on simulated video data and creating an empirical conversion between τ and *D*_lat_ (note: values >2 μm^2^·s^−1^could not be accurately determined). The *D*_lat_ values were then color-coded, using a pseudo-color look-up table, to give a visual map that could be overlaid onto the original video data (using ImageJ).

##### Super-resolution Images of M10 Molecules Moving within the Filopodium

To create a physical map of the actin bundle within the filopodium. Particle trajectories (also called particle tracks) were determined with sub-pixel resolution and plotted on top of one another to create a composite image of the track paths along the axis of the filopodia (*e.g.* Figs. 4*E* and 5).

##### Confocal Imaging of Fixed and Stained Cells

Cells that had been fixed and stained for actin using Alexa-546 phalloidin were imaged either using a Deltavision deconvolution microscope using the ×63 oil objective (for counting filopodia number) or using a Zeiss 880 LSM Airy scan confocal microscope, using a PlanApochromat ×63, 1.4NA oil immersion objective lens (Carl Zeiss Ltd., Cambridge, UK). Raw Airy scan images were deconvolved using the Zeiss Zen software to generate the images shown (Fig. 6*A*).

To count the number of filopodia, deconvolved images of whole cells taken close to the surface of the coverslip were analyzed in ImageJ. Filopodia were identified on the basis of their actin staining and the presence of Myo10 at the tip of the filopodia. Filopodia numbers, from 11 to 14 cells, were counted (4–7 cells from two separate experiments), and the data were expressed as filopodia number per cell. This approach only considers substrate-attached filopodia and not dorsal filopodia, which are challenging to estimate using a light microscopy approach.

## Author Contributions

M. P. and J. E. M. designed the research; T. G. B., T. A. N., and G. I. M. performed the research; G. I. M., M. P., T. G. B., and J. E. M. contributed new reagents/analytic tools; G. I. M., T. G. B., T. A. N., M. P., and J. E. M. analyzed the data; M. P., G. I. M., T. G. B., and J. E. M. wrote the paper. All authors reviewed the results and approved the final version of the manuscript.

## Supplementary Material

Supplemental Data

## Footnotes

4 The abbreviations used are:

TIRF: total internal reflection fluorescence
SAH: single α-helical
PH: pleckstrin homology
FERM: four-point-one ezrin-radixin-moesin
CC: coiled-coil
MSD: mean-squared displacement
FL-M10: full-length myosin 10
eGFP: enhanced GFP
PtdIns(3,4,5)P_3_: phosphatidylinositol (3,4,5)-trisphosphate
PtdIns(4,5)P_2_: phosphatidylinositol 4,5-bisphosphate.
