# Supplementary material for: A Combination of Diffusion and Active Translocation Localizes Myosin 10 to the Filopodial Tip
Source: J Biol Chem. 2016 Aug 26;291(43):22373–85. doi: 10.1074/jbc.M116.730689 (PMC5077179; doi:10.1074/jbc.M116.730689)
Supplement: Supplemental Data [file supp_291_43_22373__index.html]

A Combination of Diffusion and Active Translocation Localizes Myosin 10 to the Filopodial Tip. — A Combination of Diffusion and Active Translocation Localizes Myosin 10 to the Filopodial Tip — Myosin 10 Localizes by Active and Passive Movement — Supplemental Data 

# A Combination of Diffusion and Active Translocation Localizes Myosin 10 to the Filopodial Tip

## Supplemental Data

- Movie 1 (.avi, 43.5 MB) - MOVIE 1. HeLa cell transiently transfected with full-length, eGFP-myosin 10 (FL-M10) viewed by TIRF microscopy (37 oC).
- Movie 2 (.avi, 15.7 MB) - MOVIE 2. HeLa cell transiently transfected with FL-M10 viewed by TIRF microscopy (37 oC).
- Movie 3 (.avi, 31.2 MB) - MOVIE 3. HeLa cell transiently transfected with eGFP-myosin10 motor plus PH domains (M10-motor-PH).
- Movie 4 (.avi, 22.6 MB) - MOVIE 4. HeLa cell transiently transfected with eGFP-myosin10 isolated MyTH4-FERM isolated domains.
- Movie 5 (.avi, 3.5 MB) - MOVIE 5. HeLa cell transiently transfected with eGFP-FL-M10 moving at the base of the filopodium.
- Supplementary Movie Legends (.pdf, 40 KB) - Supplementary Movie Legends
