# Supplementary material for: A Combination of Diffusion and Active Translocation Localizes Myosin 10 to the Filopodial Tip
Source: J Biol Chem. 2016 Aug 26;291(43):22373–85. doi: 10.1074/jbc.M116.730689 (PMC5077179; doi:10.1074/jbc.M116.730689)
Supplement: Supplemental Data [file 10.1074_M116.730689_jbc.M116.730689-6.pdf]

A Combination of Diffusion and Active Translocation is Required for Myosin 10 Trafficking to the Filopodial Tip.

Thomas G. Baboolal, Gregory I. Mashanov, Tatiana A. Nenasheva, Michelle Peckham and Justin E. Molloy

SUPPLEMENTARY MOVIE LEGENDS:

MOVIE 1. HeLa cell transiently transfected with full-length, eGFP-myosin 10 (FL-M10) viewed by TIRF microscopy (37 ^{o}C). Individual molecules can be seen diffusing freely in the cell body (upper left region) and numerous filopodia can be seen toward the lower right; with single molecules trafficking towards the bright tips (seen during the first section of the video). The next section of video shows a central region of the cell with individual molecules moving at the plasma membrane, tracked by computer. Notice that molecules diffusing within the cell volume (not bound to membrane) move too rapidly to be tracked and produce a background speckled pattern. Next, a small, highly magnified region, of the cell is shown (note pixelation). The graph inset shows the accumulated power spectral density of intensity fluctuations, measured at 16 neighboring pixels, fitted to a Lorentzian function. The characteristic “roll-off” frequency allows the diffusion coefficient to be estimated (by comparison with simulated data sets and control specimens). A pseudo-color map of the local diffusion estimates is overlaid on the original video data. Finally, directed motion of FL-M10 within a single filopodium is shown and this is followed by kymograph analysis of FL-M10 movement within the same filopodium.

MOVIE 2. HeLa cell transiently transfected with FL-M10 viewed by TIRF microscopy (37 ^{o}C). This movie shows full-length, eGFP-myosin 10 molecules moving within the cell body and translocating within the filopodia. The diffusion map (determined by correlation analysis – see Movie 1 for details) is shown overlaid on the original video data.

MOVIE 3. HeLa cell transiently transfected with eGFP-myosin10 motor plus PH domains, (M10-motor-PH). The diffusion map (determined by correlation analysis – see Movie 1 for details) is shown overlaid on the original video data.

MOVIE 4. HeLa cell transiently transfected with eGFP- MyTH4-FERM isolated domains: Note the increased rate of diffusion compared to the behavior seen in previous movies.

MOVIE 5. HeLa cell transiently transfected with FL-M10 moving at the base of the filopodium. A small region of interest around the base of an individual filopodium is played back-and-forth at 10x normal speed as a video loop (please set your video playback program to “looped mode” so that the movie cycles indefinitely). Notice that the directed movement that is observed within the filopodial shaft is also apparent for a few molecules at the base, or root, of the filopodium whereas just a few microns further towards the cell body most molecules move in a chaotic and random manner with a diffusion coefficient typical of membrane bound proteins.
